# Rigorous solution for 1-D consolidation of a clay layer under haversine cyclic loading with rest period

**DOI:** 10.1186/s40064-016-3660-9

**Published:** 2016-11-17

**Authors:** Nina Müthing, Sabah S. Razouki, Maria Datcheva, Tom Schanz

**Affiliations:** 1 Chair of Foundation Engineering, Soil and Rock Mechanics, Ruhr-Universität Bochum, Bochum, Germany; 2 College of Engineering, Alnahrain University, Baghdad, Iraq; 3 Institute of Mechanics, Bulgarian Academy of Sciences, Sofia, Bulgaria

**Keywords:** Analytical solution, Consolidation, Cyclic loading, Haversine repeated loading, Rigorous solution

## Abstract

Presented in this paper is a rigorous solution of the conventional Terzaghi one-dimensional consolidation under haversine cyclic loading with any rest period. The clay deposit is either permeable at both top and bottom or permeable at the top and impermeable at the bottom. This exact analytical solution was achieved using Fourier harmonic analysis for the periodic function representing the rate of imposition of excess pore water pressure. The double Fourier series in the rigorous solution was found to be rapidly convergent. The analysis of excess pore water pressure and effective stress is done in the Matlab 2010 environment. Both the effects of rest period and frequency of cyclic loading are investigated. The analysis reveals that the excess pore water pressure arrives the steady-state at a time factor *T*_*v*_ of about 2. Furthermore, finite element method (FEM) is applied to solve numerically the corresponding consolidation problem and the FEM solution is compared to the analytical solution showing a good match.

## Background

It is well-known that cyclic loading of soils may result from natural phenomena or human activities such as wind and water waves, vehicular traffic, reciprocating machinery and others (Mitchell 1993; Zhang et al. 2009). Special structures such as silos and fluid tanks that undergo filling and discharging subject their foundation soils to loading unloading stages that repeat themselves more or less periodically over time (Conte and Troncone 2006).

Many forms of time-dependent behaviour of repeated loading such as sinusoidal, rectangular, triangular, trapezoidal and haversine waves were suggested by various authors as the type and duration of loading to be used in any repeated load test or analysis should simulate that actually occurring in the field (Zimmerer 2009; Huang 1993; Barksdale 1971; Razouki and Schanz 2011).

Zienkiewicz et al. (1980) studied a soil layer subject to a periodic surface force represented by a function in complex form containing both real and imaginary parts (Kreyszig 2006) to find out under what conditions such extremes as undrained or quasi-static assumptions can be safely used. They used their solution for earthquake analysis of an earth dam and they carried out a parametric analysis of pore pressure distribution in a seabed due to the passage of a surface wave.

Due to the fact that many problems of 1-D consolidation of cohesive soils have an equivalent problem in the heat condition in solids, it is necessary to review the wave forms considered by Carslaw and Jaeger (1959) in the field of heat diffusion. Using either the Fourier series approach or the Laplace transforms approach for solving 1-D heat diffusion problems, Carslaw and Jaeger (1959) considered, among others, periodic boundary conditions in a rectangular wave form or a sine-wave form only. This means that they focused their attention only on the homogeneous 1-D heat equation with periodic boundary conditions.

The problem of one-dimensional consolidation under cyclic loading (rectangular, triangular, sinusoidal and trapezoidal waves) has received attention by various authors, Baligh and Levadoux (1987), Favaretti and Soranzo (1995), Guan et al. (2003), Geng et al. (2006) and Hsu and Lu (2006). However, the problem of haversine repeated loading in one-dimensional consolidation analysis has received attention for the first time by Razouki and Schanz (2011). They applied a numerical implicit finite difference method to obtain the solution of the Terzaghi conventional consolidation theory under haversine cyclic loading and investigated the effect of rest period on the time variation of excess pore water pressure and effective stress. They concluded that an increase in rest period reduces the final average effective stress and hence the settlement. Razouki et al. (2013) derived an analytical solution of the Terzaghi one-dimensional consolidation under haversine cyclic loading without rest period and analysed the main features of the process based on that solution. The comparison of the analytical solution with a corresponding finite element solution shows excellent agreement.

The goal of this paper is to obtain the analytical solution in the general case of haversine cyclic loading with any rest period which is of greater importance and benefit in practice of geotechnical engineering. Haversine loading waveform with rest period to be considered here is the same as that reported by Razouki and Schanz (2011) which is a periodic function *L*(*t*) whose definition in the fundamental period is given by

1 $$L(t) = \left\{ \begin{array}{ll} 0 &\quad 0 \le t \le \dfrac{R}{2}\\ q\sin ^2\dfrac{\pi }{d}\left( t - \dfrac{R}{2}\right) &\quad \dfrac{R}{2} \le t \le d + \dfrac{R}{2}\\ 0 &\quad d + \dfrac{R}{2} \le t\le d +R \\ \end{array} \right.$$

where *q* is the amplitude of loading, *d* is the duration of loading within a loading cycle and *R* is the duration of rest period.

**Fig. 1:**
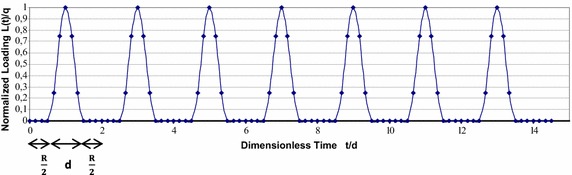
Haversine cyclic loading with rest period for the case of $\frac{R}{d}=1$

Figure 1 illustrates the form of the haversine loading function for a chosen rest period of *R*/*d* = 1. Due to the fact that only one-dimensional consolidation is of interest in this paper, the haversine loading is considered uniformly distributed and applied to the top surface of the clay deposit and as a result the loading rate is given as

2 $$\dfrac{dL}{dt} = \left\{ \begin{array}{ll} 0 &\quad 0 \le t \le \dfrac{R}{2}\\ \dfrac{q\pi }{d}\sin {\dfrac{2\pi }{d}\left( t - \dfrac{R}{2}\right) } &\quad \dfrac{R}{2} \le t \le d + \dfrac{R}{2}\\ 0 &\quad d + \dfrac{R}{2} \le t\le d +R \\ \end{array} \right.$$

## Governing differential equation and clay deposit boundary conditions

**Fig. 2:**
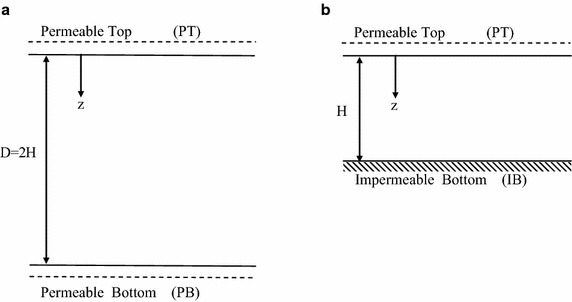
Clay layer: **a** with permeable top and bottom, **b** with permeable top and impermeable bottom

For the case of time-dependent loading, the governing differential equation for one-dimensional consolidation analysis becomes (Verruijt 1995; Coussy 2004)

3 $$\dfrac{\partial u}{\partial t} = C_z \dfrac{\partial ^{2}u}{\partial z^{2}} + \eta \,\dfrac{dL}{dt}$$

where $\eta = \dfrac{\gamma }{\gamma +\phi \,\beta },$ *γ* and *β* are the compressibility of the solid and the water and $\phi$ is the porosity; *C*_*z*_ is the coefficient of consolidation in vertical direction *z* (Fig. 2) and *u*(*z*, *t*) is the excess pore water pressure at depth *z* and time *t*. Furthermore, it is assumed that pore water is incompressible and therefore $\eta =1$.

The considered here clay deposit is a homogeneous clay layer with constant coefficients of permeability and of consolidation with a thickness of *D* = 2*H*. The top surface and the bottom surfaces are considered to be permeable (abbreviated as PTPB). Thus, the hydraulic boundary conditions are given by

4 $$u(0,t) = u(D,t) = 0$$

However, it is well-known that, for $0\le z\le H$, the solution for the PTPB case with a total thickness of 2*H* is also valid for the case of a clay layer with permeable top and impermeable bottom (PTIB) with a thickness *H* as the imposed excess pore water pressure is uniform over the whole depth of the deposit, as demonstrated in Fig. 2.

The initial condition is given as *u*(*z*, 0) = 0.

## Fourier representation of rate of imposition of the haversine load

As the rate of imposition of haversine loading given by Eq. (2) is an odd periodic function, it can be represented by a Fourier sine series as follows (Kreyszig 2006; Wylie and Barrett 1985):

5a $$\dfrac{dL}{dt} = \sum \limits _{m=1}^{\infty } b_m \sin \dfrac{2m \pi t}{R+d}$$

where

5b $$b_m = \dfrac{4}{R+d} \int \limits _\frac{R}{2}^{\frac{R+d}{2}} \dfrac{q \pi }{d} \sin \dfrac{2 \pi \left( t- \dfrac{R}{2}\right) }{d} \sin \dfrac{2m \pi t}{R+d} dt$$

It can be shown easily that after carrying out the above integration, the coefficients $b_m$ become

6 $$b_m = \dfrac{2q(R+d)}{(R+d)^2-m^2d^2} \sin \dfrac{m \pi R}{R+d}$$

Equation (6) is valid for any $\dfrac{R}{d}=\alpha$ not integer. For integer *α* the coefficient $b_{\alpha + 1}$ is of type $\dfrac{0}{0}$ and the limit at $\alpha \rightarrow m-1$ is considered in this case. Accordingly

7 $$\dfrac{dL}{dt}= \dfrac{2q(\alpha +1)}{d} \sum \limits _{m=1}^{\infty } \dfrac{ \sin \dfrac{m \pi \alpha }{\alpha +1} \sin \dfrac{2m \pi t}{d(1+\alpha )}}{(\alpha +1)^2-m^2}$$

In case of integer *α* we have

8 $$\dfrac{dL}{dt}= \dfrac{2q(\alpha +1)}{d} \sum \limits _{m=1, m \ne \alpha +1}^{\infty } \dfrac{ \sin \dfrac{m \pi \alpha }{\alpha +1} \sin \dfrac{2m \pi t}{d(1+\alpha )}}{(\alpha +1)^2-m^2} + \dfrac{\pi }{\alpha +1}\, \dfrac{q}{d}\, \sin \dfrac{2 \pi t}{d}$$

The substitution of Eq. (7) into Eq. (3) yields the following final form of the governing differential equation, in case *α* is not integer

9 $$\dfrac{\partial u}{\partial t} = C_z \dfrac{\partial ^2 u}{\partial z^2} + \dfrac{2q(1+\alpha )}{d} \sum \limits _{m=1}^{\infty } \dfrac{ \sin \dfrac{m \pi \alpha }{\alpha +1} \sin \dfrac{2m \pi t}{d(1+\alpha )}}{(1+\alpha )^2-m^2}$$

In the discussion below even the calculations are explained for non integer *α*. Therefore, the corresponding expressions for *α* integer can easily be obtained as it is explained in Eq. (8). As the excess pore water pressure *u*(*z*, *t*) is a function of both *z* and *t*, it is necessary to represent *q* = constant over the whole depth 2*H* in terms of a Fourier half-range sine series as follows

10a $$q = \sum \limits _{n=1}^{\infty } b_n \sin \dfrac{n \pi z}{2H}$$

where

10b $$b_n = \dfrac{2}{2H} \int \limits _{0}^{2H} q \sin \dfrac{n \pi z}{2H} dz = \dfrac{2 q}{n \pi } (1- \cos n \pi )$$

Thus, Eq. (9) can be written as follows

11 $$\dfrac{\partial u}{\partial t} = C_z \dfrac{\partial ^2 u}{\partial z^2} + \dfrac{4q(1+\alpha )}{ d \pi } \sum \limits _{m=1}^{\infty } \dfrac{ \sin \dfrac{m \pi \alpha }{1+\alpha } \sin \dfrac{2m \pi t}{d(1+\alpha )}}{(1+\alpha )^2-m^2} \, \sum \limits _{n=1}^{\infty } \dfrac{1- \cos n \pi }{n} \, \sin \dfrac{n \pi z}{2H}$$

## Solution of governing differential equation

### Excess pore water pressure

Since the relevant differential equation is linear, the principle of superposition of particular integrals is valid (Wylie and Barrett 1985). Thus, it is wise to obtain first of all a solution of the differential equation containing only the general term (*m*th term, in case $m \ne 1+\alpha$ ) given by $\dfrac{ \sin \dfrac{m \pi \alpha }{1+\alpha } \sin \dfrac{2m \pi t}{d(1+\alpha )}}{(1+\alpha )^2-m^2}$ in the Fourier series of *dL*/*dt* in Eq. (11), which leads to the following partial differential equation

12 $$\dfrac{\partial u}{\partial t} = C_z \dfrac{\partial ^2 u}{\partial z^2} + \dfrac{4q(1+\alpha )}{d \pi } \dfrac{ \sin \dfrac{m \pi \alpha }{1+\alpha } \sin \dfrac{2m \pi t}{d(1+\alpha )}}{(1+\alpha )^2-m^2} \, \sum \limits _{n=1}^{\infty } \dfrac{1- \cos n \pi }{n} \, \sin \dfrac{n \pi z}{2H}$$

To arrive at a solution of the above differential equation that satisfies the hydraulic boundary conditions given by Eq. (4), the following series solution is introduced

13 $$u(z,t) = \sum \limits _{n=1}^{\infty } T_n(t) \sin \dfrac{n \pi z}{2H}$$

It can be shown easily that the substitution of Eq. (13) into Eq. (12) yields the following first order ordinary differential equation

14 $$\dfrac{dT_n}{dt} + \dfrac{n^2 \pi ^2 C_z}{4H^2} T_n = \dfrac{4q(1+\alpha ) (1-\cos n \pi ) \sin \dfrac{m \pi \alpha }{1+\alpha } }{n d \pi [(1+\alpha )^2-m^2]}\,\sin \dfrac{2m \pi t}{d(1+\alpha )}$$

According to Wylie and Barrett (1985), the general solution of this ordinary differential equation can be written as follows

15 $$T_n(t) = \dfrac{4q(1+\alpha )(1- \cos n \pi ) \sin \dfrac{m \pi \alpha }{1+\alpha }}{n d \pi \left( (1+\alpha )^2-m^2\right) } \left( \int \sin \dfrac{2m \pi t}{d(1+\alpha )} \exp \left( \dfrac{n^2 \pi ^2 T_v}{4}\right) dt + K_n \right) \, \exp \left( -\,\dfrac{n^2 \pi ^2 T_v}{4}\right)$$

where $T_v = \dfrac{C_z\,t}{H^2}$ (dimensionless) and *K*_*n*_ is a constant.

After carrying out the required integration, Eq. (15) can be written as follows

16 $$\begin{aligned} T_n(t) &= \dfrac{4q(1+\alpha )(1- \cos n \pi ) \sin \dfrac{m \pi \alpha }{1+\alpha }}{n \pi [(1+\alpha )^2-m^2]} \, \dfrac{4n^2\,T_0(1+\alpha )^2 }{64m^2+n^4 \pi ^2T^2_0(1+\alpha )^2} \, \left( \sin \dfrac{2m\pi t}{d(1+\alpha )}- \dfrac{8m \cos \dfrac{2m\pi t}{d(1+\alpha )}}{n^2\pi nT_0\left( 1+ \alpha \right) }\right) \\ &\quad +\,K_n \exp \left( -\dfrac{n^2 \pi ^2T_v}{4}\right) \end{aligned}$$

where

17 $$T_0 = \dfrac{C_zd}{H^2}$$

The constant *K*_*n*_ can be determined from the initial condition, that means *u*(*z*, 0) = 0 and hence *T*_*n*_(0) = 0, yielding

18 $$K_n = \dfrac{128qm(1+\alpha )(1- \cos n \pi )(1+ \alpha ) \sin \dfrac{m \pi \alpha }{1+\alpha }}{n \pi ^2[(1+\alpha )^2-m^2][64m^2+n^4 \pi ^2 T^2_0 (1+ \alpha )^2]}$$

The substitution of Eq. (18) into Eq. (16) yields

19 $$T_n=\dfrac{16qn(1+ \alpha )^3(1-\cos n\pi )T_0 \sin \dfrac{m \pi \alpha }{1+\alpha }}{\pi [(1+ \alpha )^2-m^2][64m^2+\pi ^2 n^4T^2_0(1+ \alpha )^2]}\left( \sin \dfrac{2m\pi t}{d(1+\alpha )}-\dfrac{8m\cos \dfrac{2m\pi t}{d(1+\alpha )}}{n^2\pi T_0(1+ \alpha )}+\dfrac{8m\exp \left(-\dfrac{n^2\pi ^2 T_v}{4}\right)}{n^2\pi T_0(1+ \alpha )}\right)$$

The substitution of Eq. (19) into Eq. (13) taking into account $\sum \nolimits _{m=1}^{\infty }$ in Eq. (11), the excess pore water pressure *u*(*z*, *t*) becomes

20 $$u(z,t)=\dfrac{32q(1+\alpha )^3T_0}{\pi }\sum \limits _{m=1}^{\infty } \dfrac{\sin \dfrac{\alpha m\pi }{1+\alpha }}{(1+\alpha )^2-m^2} \sum \limits _{k=0}^{\infty }C_{m,2k+1}$$

with

$$C_{m,2k+1}= \dfrac{(2k+1) \left\{ \sin \dfrac{2m\pi T_v}{T_0(1+\alpha )}-\dfrac{8m}{(2k+1)^2\pi T_0(1+\alpha )} \left( \cos \dfrac{2m\pi T_v}{T_0(1+\alpha )}-\exp \left( -\dfrac{(2k+1)^2\pi ^2T_v}{4}\right) \right) \right\} \sin \dfrac{(2k+1)\pi z}{2H}}{64m^2+\pi ^2(2k+1)^4T^2_0(1+\alpha )^2}$$

**Fig. 3:**
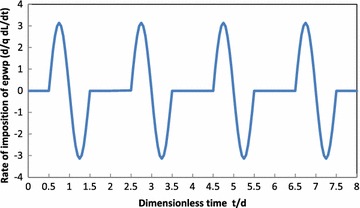
Time variation of rate of imposition of excess pore water pressure (epwp) for the case of haversine cyclic loading for *T*_0_ = 0.15

Figure 3 shows exemplarily the time variation of rate of imposition of excess pore water pressure (epwp) for the case of haversine cyclic loading for *T*_0_ = 0.15.

For the special case of zero rest period (i.e. *α* = 0), all the terms in the first series $\sum \limits _{m=1}^{\infty }\dfrac{\sin \dfrac{\alpha m \pi }{1+\alpha }}{(1+\alpha )^2-m^2}$ in Eq. (20) vanish except the first term for *m* = 1. The limit of this term for $\alpha \rightarrow 0$ is $\lim \limits _{\alpha \rightarrow 0}\dfrac{\sin \dfrac{\alpha \pi }{1+\alpha }}{(1+\alpha )^2-1} = \dfrac{\pi }{2}$

The substitution of this fact into Eq. (20) yields

21 $$u(z,t)=16q\sum \limits _{k=0}^{\infty }\dfrac{(2k+1)T_0}{64+(2k+1)^4\pi ^2T_0^2}\left[ \sin \dfrac{2\pi t}{d}-\dfrac{8}{(2k+1)^2\pi T_0}{\cos \dfrac{2\pi t}{d}-\exp \left( -\dfrac{(2k+1)^2\pi ^2 T_v}{4}\right) }\right] \sin \dfrac{(2k+1)\pi z}{2H}$$

This solution is exactly that presented by Razouki et al. (2013).

### Effective stress

To arrive at the time variation of effective vertical stress at any depth in the clay deposit, both the total stress and the excess pore water pressure should be known. For this purpose, the loading function, given by Eq. (1), representing the total vertical stress $\sigma (t)$, should be expressed in terms of a Fourier cosine series as it is an even periodic function. Thus

22 $$L(t)=\dfrac{a_0}{2}+\sum \limits _{n=1}^{\infty } a_0 \cos \dfrac{2\,n \pi \, t}{R+d}$$

According to Wylie and Barrett (1985), *a*_0_ and *a*_*n*_ can be calculated as follows

23a $$a_0= \dfrac{4q}{R+d} \int \limits _{\frac{R}{2}}^{\frac{R+d}{2}} \sin ^2 \pi (\dfrac{t}{d}-\dfrac{R}{2d}) dt = \dfrac{q}{1+\alpha }$$

23b $$a_n= \dfrac{4q}{R+d} \int \limits _{\frac{R}{2}}^{\frac{R+d}{2}} \sin ^2 \pi \left( \dfrac{t}{d}-\dfrac{R}{2d}\right) \cos \dfrac{2 n \pi t}{R+d} dt = -\dfrac{q(1+ \alpha )^2 \sin \dfrac{\alpha n \pi }{1+\alpha }}{n \pi [(1+ \alpha )^2 - n^2]}$$

And, the Fourier series for the loading function given by Eq. (22) becomes

24 $$L(t) = q\left\{ \dfrac{1}{2(1+\alpha )}-\dfrac{(1+\alpha )^2}{\pi }\sum \limits _{n=1}^{\infty }\dfrac{\sin \dfrac{\alpha n \pi }{1+\alpha }}{n[(1+\alpha )^2-n^2]} \cos \dfrac{2n\pi \dfrac{T_v}{T_0}}{1+\alpha }\right\}$$

Thus, the effective vertical stress $\sigma '(z,t)$ for any value of *α* becomes

25 $$\sigma '(z,t) = L(t) - u(z,t)$$

where *L*(*t*) is given by Eq. (24) and *u*(*z*, *t*) is given by Eq. (20).

## Verification and parametric study

For the purpose of calculating the time variation of excess pore water pressure at any depth in the clay deposit based on the analytical solution, a computer program using the software Matlab2010 was written. To achieve high accuracy of results, one hundred terms of each series was covered through the analysis to ensure convergence of each infinite series. Moreover, the analytical solution was compared to the approximate numerical solution obtained via finite element method (FEM) employing the FE code PLAXIS.

### Effect of rest period

To study the effect of rest period on the consolidation process due to repeated haversine loading, the solution was obtained for the PTIB case and the following *α*-values of *α* = 1, 2, 3 and 4.

**Fig. 4:**
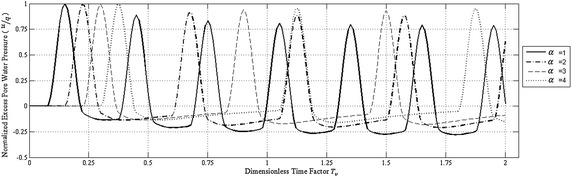
Effect of rest period on the time variation of normalized excess pore water pressure at the impermeable bottom of PTIB clay deposit for *T*_0_ = 0.15

Figure 4 shows the effect of rest period on the time variation of normalized excess pore water pressure at the impermeable bottom of the PTIB clay deposit for *T*_0_ = 0.15. It is quite obvious from this figure that for each *α*-value, the negative excess pore water pressure increases in absolute value with dimensionless time factor causing the positive excess pore water pressure to decrease and the effective stress to increase converging in average to its final value.

**Fig. 5:**
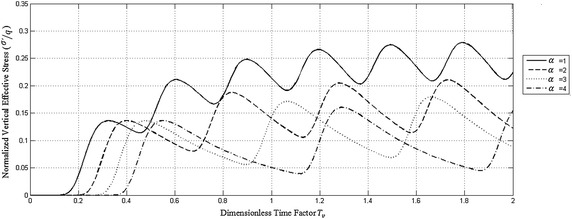
Effect of rest period on the time variation of normalized vertical effective stress at the impermeable bottom of PTIB clay deposit for *T*_0_ = 0.15

Figure 5 shows the effect of rest period (*α*-values) on the normalized effective stress at the impermeable bottom of the deposit. It can be seen from this figure that for each *α*-value the average normalized effective stress converges to the actual average of the applied cyclic loading.

The average of the applied normalized cyclic loading is 0.25, 0.167, 0.125 and 0.1 for *α* = 1, 2, 3 and 4 respectively.

Accordingly, Fig. 5 shows the decrease in effective stress due to increase in *α*-values. This means that an increase in the rest period for the applied haversine cyclic loading causes a decrease in the settlement of the deposit.

### Effect of frequency of cyclic loading

To study the effect of frequency of cyclic loading on the consolidation process, the solution was obtained for the PTIB case of *α* = 1 for the following *T*_0_ values namely *T*_0_ = 0.5, 1, 2, 5 and 10.

**Fig. 6:**
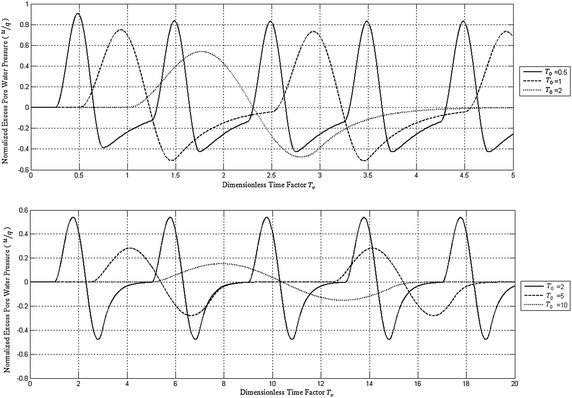
Effect of rest period on the time variation of normalized excess pore water pressure at the impermeable bottom of PTIB clay deposit for α = 1 and various *T*_0_

**Fig. 7:**
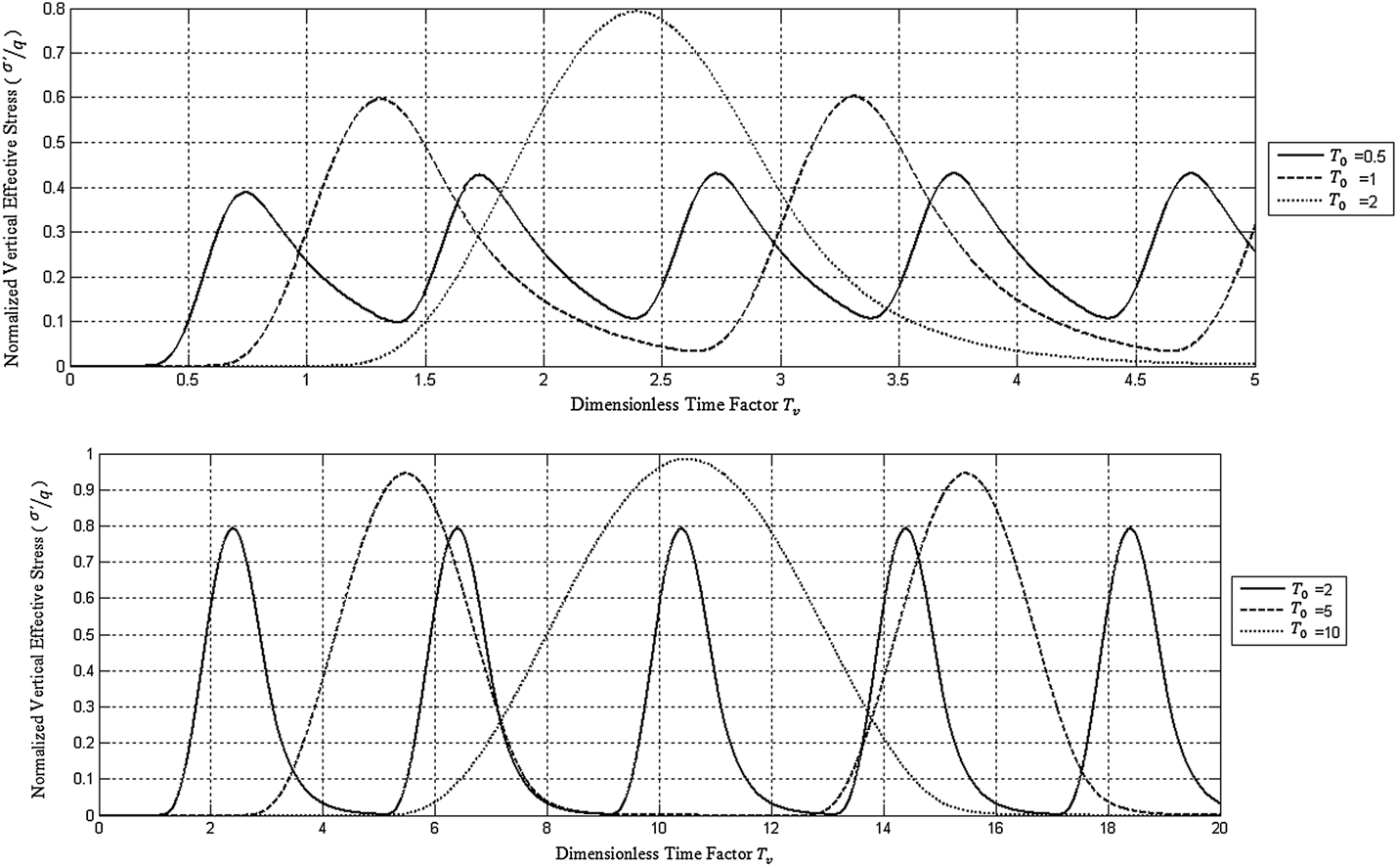
Effect of rest period on normalized effective stress at the impermeable bottom of PTIB clay deposit for α = 1 and various *T*_0_

Figure 6 shows the effect of frequency of loading (*T*_0_ values) on the normalized excess pore water pressure at the impermeable bottom of the clay deposit. It is quite obvious from this figure that for each To value, the negative excess pore water pressure decreases in absolute value causing an increase in effective stress. Similarly, Fig. 7 shows the effect of frequency of cyclic loading on the effective stress at the bottom of the clay deposit. It can be concluded that the effective stress increases with time but with some fluctuations without changing sign. As *T*_0_ increases the maximum effective stress also increases converging to a steady state after few numbers of cycles.

## Comparison with the results of analysis via finite element method (FEM)

The analytical solution was compared with the numerical solution of the considered consolidation problem obtained using the finite element (FE) software PLAXIS (Brinkgreve et al. 2010). However, the problem to be solved with help of the FEM is posed slightly differently. To avoid a singular system of equations, a finite stiffness is assigned to the water. By combining the linear elastic behaviour of the soil skeleton and the flow of the water through the pore system a system of equations for displacements and pore pressure as unknowns in the FE nodes is defined. Details of the numerical model can be found in Razouki et al. (2013).

**Fig. 8:**
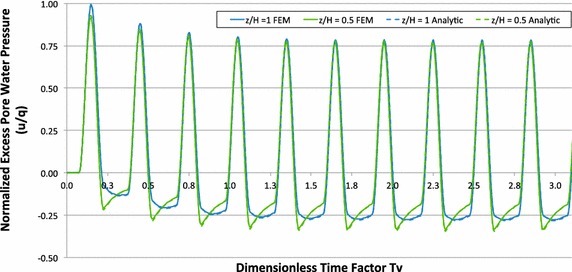
Pore water pressure dissipation over sample height

**Fig. 9:**
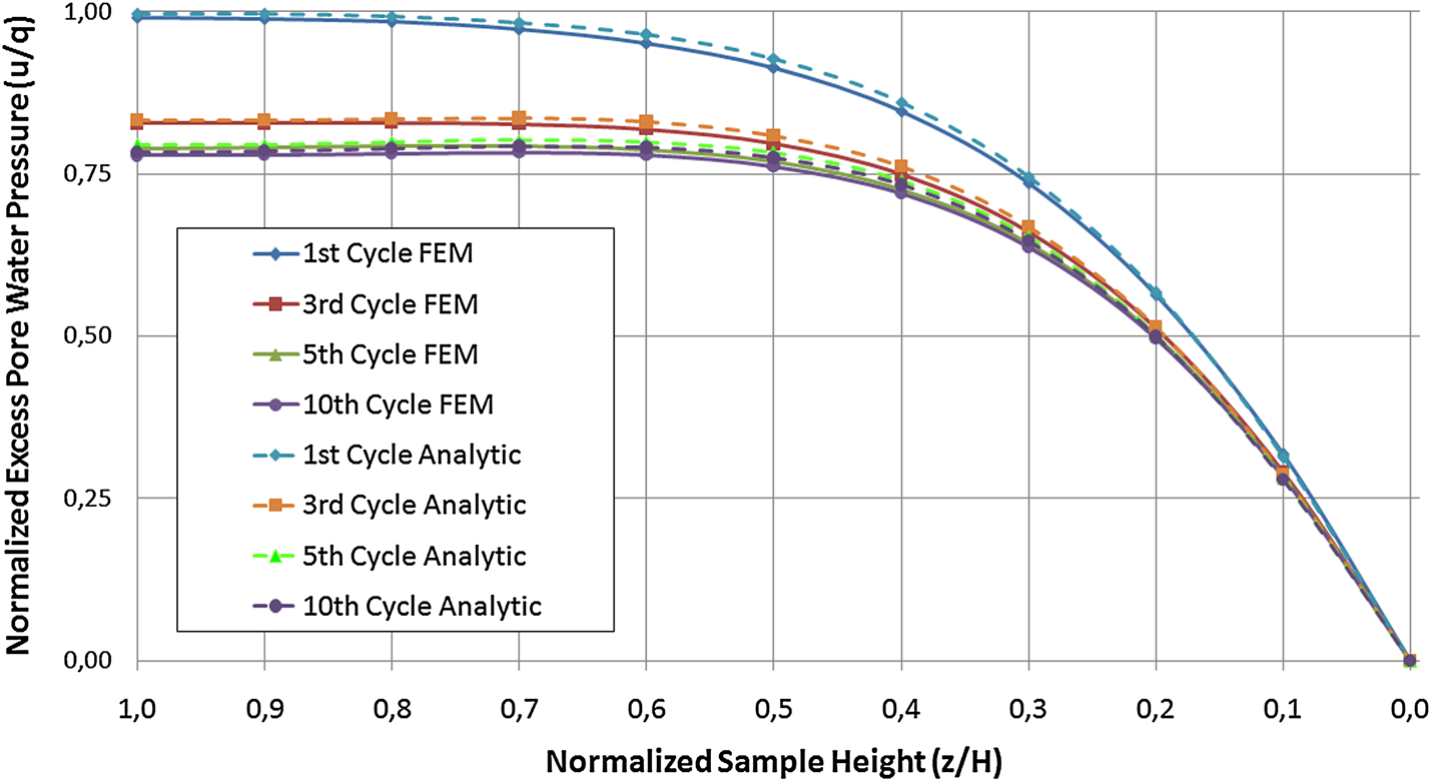
Pore water pressure over sample height for different loading cycles (at time of maximum loading)

In Fig. 8 the excess pore water pressure dissipation at two locations over the sample height (bottom and centre) obtained analytically and numerically is compared for *T*_*v*_ = 0.15 and *α* = 1. After approximately ten cycles the pore water pressure reaches the quasi steady state. Only for the first few cycles there exists a difference in the maximum pore water pressure in both, the middle and bottom of the model. With increasing the number of cycles this difference almost vanishes. Figure 9 provides information to study in details the time to reach the quasi steady state. It depicts the results for *u*/*q* versus *H* obtained via analytical and FE methods, considering the maximum loading at time *d*/2. The discrepancy in pore water pressure distribution vanishes for about ten cycles. The two solutions fit well.

Summarizing the results presented in this section it can be concluded that the analytical and numerical solutions coincide perfectly at least for pore water pressure and effective stress evolution at the considered three locations and the fit remains good for different rest periods and load frequencies.

## Conclusions

The main conclusions of this work can be summarized as follows:

- Although the loading function for imposed excess pore-water is always positive, the excess pore water pressure at any depth in the clay deposit (PTPB or PTIB case) changes sign during the consolidation process causing both positive and negative “excess” pore-water pressure to develop.
- Due to the haversine cyclic loading, the transient state of the consolidation process is almost completed at a dimensionless time factor *T*_*v*_ of about 2.0. Therefore, the steady state of undamped oscillation takes place and continues with continuous cyclic loading.
- For a given dimensionless time factor *T*_0_, an increase in the rest period causes the final average effective stress to decrease and to converge to the average of the loading function. For *T*_0_ = 0.15 and *α* = 1, 2, 3 and 4, the average effective stress converges to $\frac{q}{4}$, $\frac{q}{6}$, $\frac{q}{8}$, and $\frac{q}{10}$ respectively. The decrease in effective stress causes the settlement of the deposit to decrease.
- A decrease in the frequency of the loading function (*T*_0_ increases) causes the maximum effective stress to increase converging to a steady state after a few number of cycles.
